# Spatiotemporal dynamics of the Southern California Asian citrus psyllid (*Diaphorina citri*) invasion

**DOI:** 10.1371/journal.pone.0173226

**Published:** 2017-03-09

**Authors:** Brett R. Bayles, Shyam M. Thomas, Gregory S. Simmons, Elizabeth E. Grafton-Cardwell, Mathew P. Daugherty

**Affiliations:** 1 Department of Entomology, University of California Riverside, Riverside, California, United States of America; 2 School of Health and Natural Sciences, Dominican University of California, San Rafael, California, United States of America; 3 United States Department of Agriculture, Animal and Plant Health Inspection Service, Salinas, California, United States of America; Fujian Agriculture and Forestry University, CHINA

## Abstract

Biological invasions are governed by spatial processes that tend to be distributed in non-random ways across landscapes. Characterizing the spatial and temporal heterogeneities of the introduction, establishment, and spread of non-native insect species is a key aspect of effectively managing their geographic expansion. The Asian citrus psyllid (*Diaphorina citri*), a vector of the bacterium associated with huanglongbing (HLB), poses a serious threat to commercial and residential citrus trees. In 2008, *D*. *citri* first began expanding northward from Mexico into parts of Southern California. Using georeferenced *D*. *citri* occurrence data from 2008–2014, we sought to better understand the extent of the geographic expansion of this invasive vector species. Our objectives were to: 1) describe the spatial and temporal distribution of *D*. *citri* in Southern California, 2) identify the locations of statistically significant *D*. *citri* hotspots, and 3) quantify the dynamics of anisotropic spread. We found clear evidence that the spatial and temporal distribution of *D*. *citri* in Southern California is non-random. Further, we identified the existence of statistically significant hotspots of *D*. *citri* occurrence and described the anisotropic dispersion across the Southern California landscape. For example, the dominant hotspot surrounding Los Angeles showed rapid and strongly asymmetric spread to the south and east. Our study demonstrates the feasibility of quantitative invasive insect risk assessment with the application of a spatial epidemiology framework.

## Introduction

Human-mediated changes to ecosystems can significantly impact the health and well-being of plant, animal, and human populations [1]. Biological invasions are an important aspect of this global change and are accelerating in many regions where human activities facilitate introduction events [2, 3]. Urban environments play a particularly important role in the establishment of invasive insect species by serving as refuges and, ultimately, sources of invasive species spread [4–7]. This is especially true in regions where extensive urban encroachment may strengthen further linkages with nearby agricultural or natural habitats [8].

California has faced an influx of phytophagous insects that are important pests in agricultural and natural systems, yet whose establishment is centered in urban and suburban areas. Recent examples include the brown marmorated stink bug (*Halyomorpha halys*), bagrada bug (*Bagrada hilaris*), red palm weevil (*Rhynchophorus ferrugineus*), and polyphagous shot hole borer (Euwallacea spp.) [9]. These invaders threaten landscape flora, and put adjacent habitats at risk in the event of a spillover. Of particular concern agriculturally is the detection in 2008 of the Asian citrus psyllid (*Diaphorina citri*), a vector of the bacterium (*Candidatus* Liberibacter asiaticus and other spp.) that is associated with huanglongbing (HLB) or citrus greening disease. *D*. *citri* is likely native to Southeast Asia or India, but has spread to the Middle East, South America, Central America, and more recently, Mexico and the United States [10, 11]. This psyllid specializes on plants in the family Rutaceae, but feeds broadly within the family on different citrus varieties and many close relatives. HLB disease is characterized by mottled leaves, deformed and off-flavor fruit, defoliation and plant death [12, 13]. No citrus varieties are fully resistant to HLB and, to date, no cure for infected trees is available. As a result, regions where the co-occurrence of *D*. *citri* and HLB have become widespread (i.e. Florida) have suffered substantial economic losses [14]. Therefore, it is critical to limit pathogen spread by controlling *D*. *citri* populations, particularly in California and other regions where the disease is not yet widespread.

The Asian citrus psyllid was first documented in California in a residential setting in San Diego County in 2008. In response, a range of measures were implemented in an attempt to contain its spread [15]. These included establishment of an extensive monitoring program, the establishment of quarantines and insecticide treatments to limit human-assisted movement of the insect via nurseries and harvested fruit, as apparently occurred in Florida [16], protective structures to house nursery stock, residential and commercial insecticide treatment programs, and starting in 2011 a biological control program employing the nymphal parasitoid *Tamarixia radiata* for residential areas [17]. Despite these management efforts, *D*. *citri* continued its spread throughout Southern California. In 2012, the first case of HLB was documented in a residential area of Los Angeles County [15, 18].

**Fig 1:**
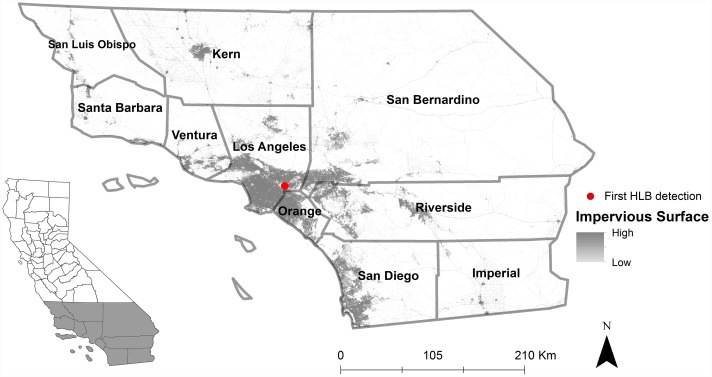
Study area including ten Southern California counties.

The progressive stages of biological invasions (introduction, establishment, spread, and impact) are governed by spatial and temporal processes that tend to be distributed in non-random ways across landscapes [19–21]. In California, a convergence of social and ecological factors appear to be contributing to the establishment of *D*. *citri* [22], yet little is published about the dynamic spread of *D*. *citri* in this region. Using georeferenced *D*. *citri* occurrence data from an area-wide network of trapping from 2008–2014, we sought to characterize the extent and features of the geographic expansion of this invasive vector species as a step towards understanding and mitigating the factors driving its invasion. Leveraging a suite of spatial statistics, our objectives were to: (1) describe the spatial distribution and temporal dynamics of *D*. *citri* in Southern California; (2) identify the locations of statistically significant *D*. *citri* hotspots; and (3) quantify the extent of anisotropic (i.e. directionally-dependent) spread throughout the Southern California study area (Fig 1).

## Materials and methods

### *D*. *citri* monitoring data source

We obtained a database of georeferenced *D*. *citri* collection sites throughout Southern California from 2008 through 2014 from the California Department of Food and Agriculture (CDFA). These include upwards of 78,000 *D*. *citri* detection records from 14 x 23 cm double-sided yellow panel traps that were deployed throughout ten counties in the Southern part of California (i.e. San Luis Obispo, Santa Barbara, Ventura, Los Angeles, Kern, San Bernardino, Riverside, Imperial, San Diego, and Orange) (Fig 1). Records come from multiple *D*. *citri* monitoring programs being conducted in parallel in these counties, totaling collectively up to more than 5,000 unique trapping locations per county over the year, with traps checked on average once per month, but up to three times per month.

The *D*. *citri* trapping program was intended primarily to demarcate the geographic extent of *D*. *citri* distribution to inform the establishment of quarantine zones and prioritize management options. Given this intent, there are important implications for the nature of the data that were available for our analyses. First, data on the geographic location of insect presence was limited to *D*. *citri* positive sampling sites (i.e. *D*. *citri* presences) rather than absences as well. In addition, since the number of *D*. *citri* adults collected on a trap was not reported consistently, our data only considers *D*. *citri* positive traps rather than counts per trap (i.e. traps with one or more *D*. *citri* caught during a given census were considered equivalent presences). Finally, the locations of traps and trapping effort in certain areas shifted over time (Table A in S1 File). Despite these potential limitations with the data, we expect that the sheer volume of *D*. *citri* positive records in the database (i.e. more than 78,000 positive traps over 7 years) allows for robust description of the broad dynamics of *D*. *citri* in the region.

**Fig 2:**
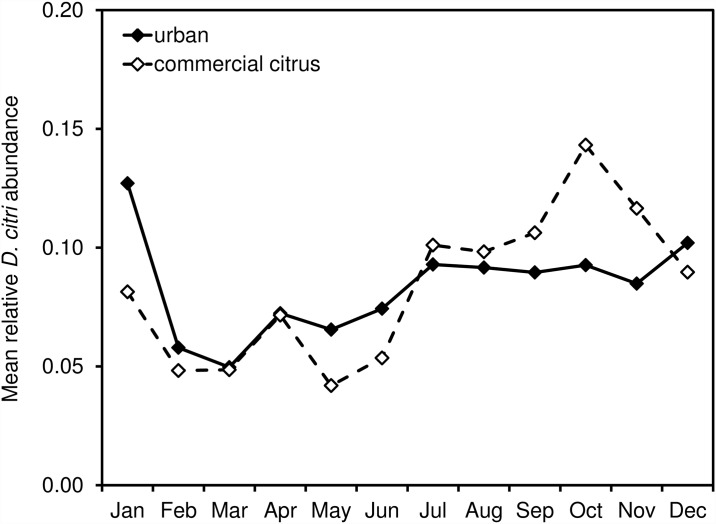
Cumulative annual *D*. *citri* occurrences in urban areas versus commercial citrus.

For this study we largely restricted analyses to those data from urban and suburban areas (hereafter referred to collectively as “urban”) in Southern California. This is primarily due to the vast majority of *D*. *citri* detections over the first five years of the program occurring in these areas (i.e. > 95%) as opposed to commercial citrus (Fig 2). To better delineate gradients of *D*. *citri* abundance and to conduct spatial analyses, we aggregated the number of *D*. *citri* positive traps into 5 x 5 km grid cells. Prior to estimating abundance, the data were checked for identical georeferenced records in a given year, and all duplicate records were deleted. Unless otherwise noted the response variable considered in analyses was cumulative count of positive *D*. *citri* traps per grid cell per unit time (i.e. *D*. *citri* abundance).

### Spatial analysis

To assess whether a geographic pattern (i.e. clustered, random, or evenly dispersed) of *D*. *citri* positive sites existed during the study period, we utilized the global Moran’s *I* measure of spatial autocorrelation. Specifically, we computed the incremental Moran’s *I* statistic for each year to detect the peak distance at which spatial clustering of the *D*. *citri* data was maximized (Table B in S1 File). These statistically significant peak distances were then used as distance thresholds for subsequent local spatial statistical tests. We used several locally-derived spatial statistics to assess whether the observed spatial pattern of *D*. *citri* during each year was significantly different from a distribution that would be expected if underlying spatial processes were random. Both the local Moran’s *I* and Getis-Ord Gi* were calculated as complementary measures of the extent to which *D*. *citri* counts in neighboring grid cells were similar or dissimilar. To further complement these purely spatial measures, we added a temporal aspect to the analysis by calculating the Kulldorff space-time permutation scan statistic. Clusters of higher than expected *D*. *citri* positive sampling sites may be considered ‘hotspots’ of elevated entomological risk [23].

First, we computed the local Moran’s *I* statistic, which uses a measure of local spatial autocorrelation at each location, as a means of detecting the geographic location of hotspots [24]. Significant local Moran’s *I* clusters represent areas of similarly high or low numbers of *D*. *citri* positive collection sites. We aggregated grid cell clusters as “high-high” hotspots of similarly elevated *D*. *citri* abundance (high risk cells next to other high risk cells) or “low-low” cold spots of similarly lower *D*. *citri* abundance (low risk cells next to other low risk cells). In addition, the local Moran’s *I* statistic produces measures of outliers, including indicators of high risk cells next to low risk cells (“high-low” outliers) and low risk cells next to high risk cells (“low-high” outliers). Next, we calculated the Getis-Ord Gi* statistic for each grid cell to identify locations where the magnitude of *D*. *citri* abundance, compared to other parts of the study area, was higher than would be expected by chance [25, 26]. Finally, we implemented the Kulldorff space-time permutation statistic using SaTScan spatial software to identify statistically significant space-time clusters (i.e. space-time hotspots) across each year of the study period [27]. Space-time clusters, defined as an excess of *D*. *citri* positive sites that are close to one another in both space and time, were identified by a cylindrical scanning window with a circular geographic base and height defined by a 1-year time interval. To test the null hypothesis that all *D*. *citri* positive sites are independent of space-time interaction, a test statistic was derived by computing maximum likelihood ratios and corresponding significance values obtained through 999 Monte Carlo simulations. The scan test reports the relative risk (RR) of each significant space-time cluster, which is estimated as the observed number of *D*. *citri* positive sites divided by the expected number of sites within the cluster. We used the magnitude of the test statistic to rank each statistically significant cluster. Spatial analysis was conducted using ArcGIS v.10.2.2 [28] and the SaTScan spatial software program [29].

### Quantifying anisotropic spread

To quantify the extent of geographic dispersion of *D*. *citri* across time, we modeled the spread of *D*. *citri* positive sites, as well as the spread of the statistically significant hotspots. This consisted of two complimentary analyses.

First, kernel density raster maps were developed using *D*. *citri* abundance within grid cells for 7 incremental time periods: 2008, 2008–09, 2008–10, 2008–11, 2008–12, 2008–13 and 2008–14. Kernel density estimation is a smoothing technique used to capture the intensity or magnitude of a given spatial event within a study region. To identify the appropriate search radius needed for smoothing, we first analyzed the *D*. *citri* abundance data for each of the incremental time periods for spatial autocorrelation. The resulting kernel density rasters were then classified using the standard deviation scheme into 7 bands of varying density range values for the dominant Los Angeles hotspot. The kernel density raster maps (S1 Fig) were then used to capture the rate of spread in each of the cardinal and inter-cardinal directions (i.e. N, E, W, S, NE, SE, NW, and SW). To quantify spread rate, the distance covered by *D*. *citri* in a given time period was estimated relative to the earliest known occurrence of *D*. *citri* in Los Angeles County. For each direction the rate of spread was quantified as the slope of the regression between cumulative number of years of *D*. *citri* invasion since 2009 and distance covered by *D*. *citri* density raster from the hypothetical point of origin in each incremental time period.

To further quantify the extent of directionally-dependent spread, standard deviational ellipses (SDE) were calculated to summarize the central tendency, dispersion and directional trends for both *D*. *citri* abundance per grid cell and hotspots identified with the Getis-Ord Gi* statistic. Each SDE represent areas of higher concentrations of *D*. *citri* positive sampling sites or presence of hotspots and provide measures of the extent of asymmetrical distribution of the data across the landscape [30]. We calculated SDE for each year in the study period using yearly *D*. *citri* distribution data throughout Southern California.

## Results

### General spatial and temporal patterns

**Fig 3:**
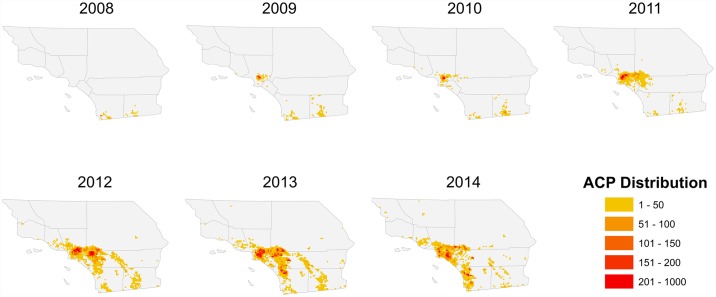
Cumulative *D*. *citri* occurrences in Southern California, 2008–2014.

**Fig 4:**
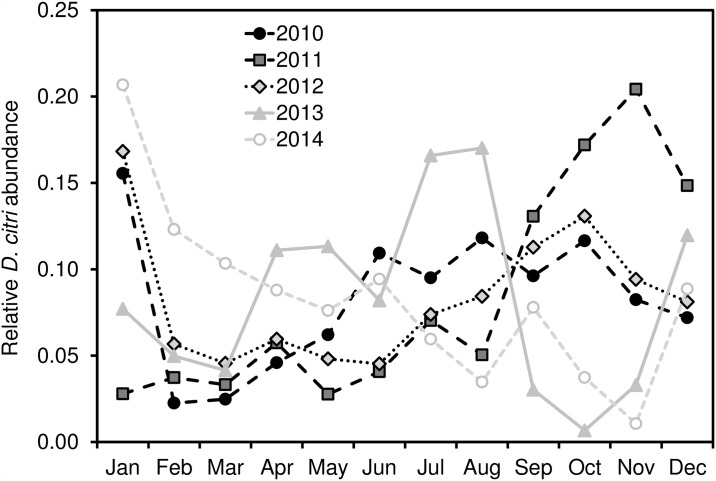
Proportion of annual urban *D*. *citri* occurrences in a given month. Only those years shown for which there were adequate occurrences each month of the year (2010–2014 for urban, 2013–2014 for commercial citrus).

**Fig 5:**
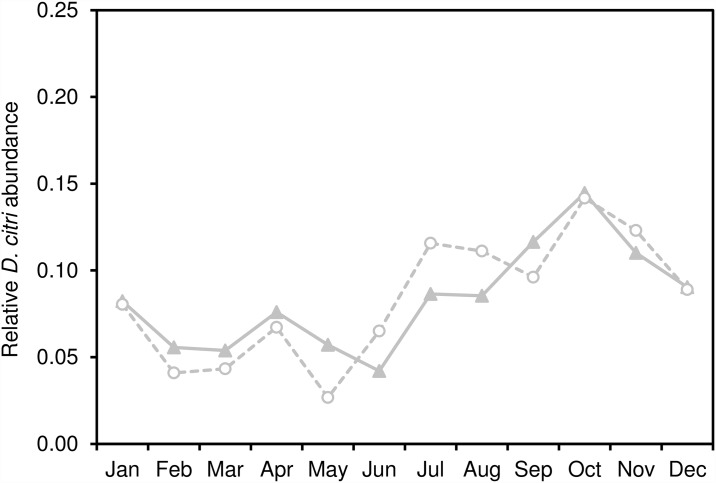
Proportion of annual commercial *D*. *citri* occurrences in a given month. Only those years shown for which there were adequate occurrences each month of the year (2010–2014 for urban, 2013–2014 for commercial citrus).

Since 2008, the cumulative abundance of *D*. *citri* in Southern California increased dramatically, particularly in the urban environment (Fig 2). After first appearing on residential citrus in San Diego County, *D*. *citri* positive sites were subsequently identified in parts of Imperial, Orange, Los Angeles, Riverside, San Bernardino, Ventura, and eventually Santa Barbara, San Luis Obispo and Kern Counties (Fig 3). Within these areas, the geographic focus of *D*. *citri* was primarily urban, especially in the first 5 years of the invasion, with the first detections in commercial citrus groves in 2011. For urban areas, we found equivocal evidence of seasonality over the study period, with a general increase in the frequency of *D*. *citri* detections during the fall months; however, the year to year distribution was variable and largely asynchronous (Fig 4). In the two years for which the frequency of commercial psyllid finds was sufficient to analyze (i.e. 2013, 2014), these areas demonstrated a more pronounced increase in the fall months, likely attributable to onset of fall citrus flush (Fig 5). Neither urban nor commercial citrus showed clear evidence of a spring peak in *D*. *citri* positive traps, a time of year that is typically associated with the largest peak in citrus flushing in California.

### Hotspots and space-time clusters

**Fig 6:**
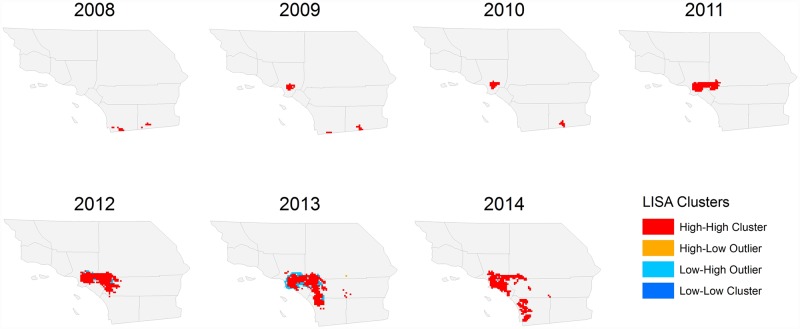
Local Moran’s *I* clusters of *D*. *citri* abundance, 2008–2014.

**Fig 7:**
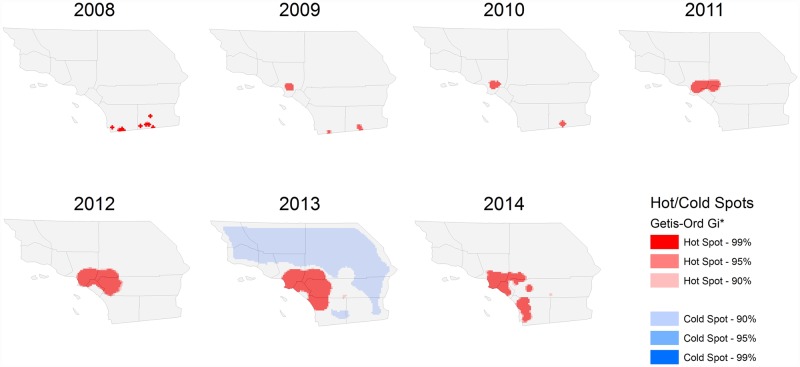
Hot and cold spots of *D*. *citri* abundance, 2008–2014.

We found evidence that the spatial distribution of *D*. *citri* in Southern California during the 7 year time period was non-random. Significant measures of spatial autocorrelation in each year indicated clustering of *D*. *citri* positive sites. We identified the location of these clusters and documented their spread using several local statistics. In 2008, both local Moran’s *I* (Fig 6) and Getis-Ord Gi* (Fig 7) statistics indicated that *D*. *citri* hotspots were located near the United States-Mexico ports of entry in San Diego and Imperial Counties. In 2009, the first *D*. *citri* hotspot was detected within an urbanized area of Los Angeles County. Subsequently, the dominant hotspot surrounding Los Angeles showed rapid and strongly asymmetric spread to the south and east. Following the establishment of the Los Angeles hotspot, clusters of high *D*. *citri*-abundance appeared to the south and east in Orange, San Bernardino, and Riverside Counties. Although *D*. *citri* detections in San Diego and Imperial counties continued to occur throughout the study period (Fig 3), the statistical significance of hotspots in this region appears to have been weakened relative to the magnitude of the Los Angeles hotspots in subsequent years.

**Fig 8:**
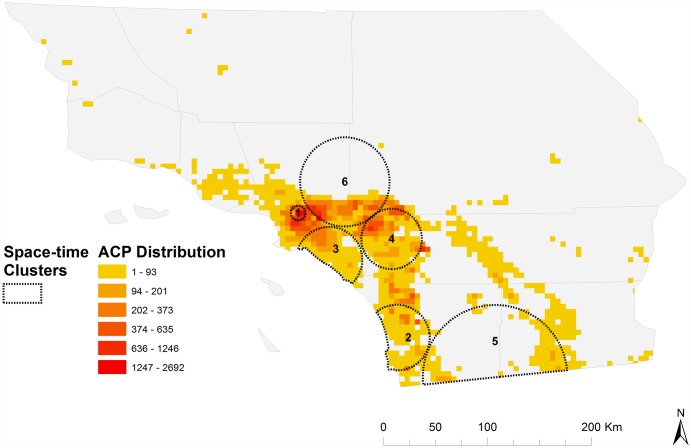
Space-time clusters depicting anisotropic spread of *D*. *citri* hotspots.

We identified six distinct space-time clusters of elevated *D*. *citri* abundance that were ranked from 1 to 6 based on decreasing relative risk measures (Table C in S1 File). Four probable clusters (clusters 1, 3, 4, 6) were located in Los Angeles, Orange, San Bernardino, and Riverside Counties and two (clusters 2, 5) in San Diego and Imperial Counties (Fig 8). The first, and most likely, cluster to be detected during the study period (cluster 5) was located on the San Diego-Mexico border in San Diego and Imperial County in 2008. Los Angeles space-time clusters were detected in 2009, with a small geographic cluster of elevated risk (RR = 4.08, p<0.001) occurring in the downtown region from 2009–2011.

### Anisotropic spread

**Fig 9:**
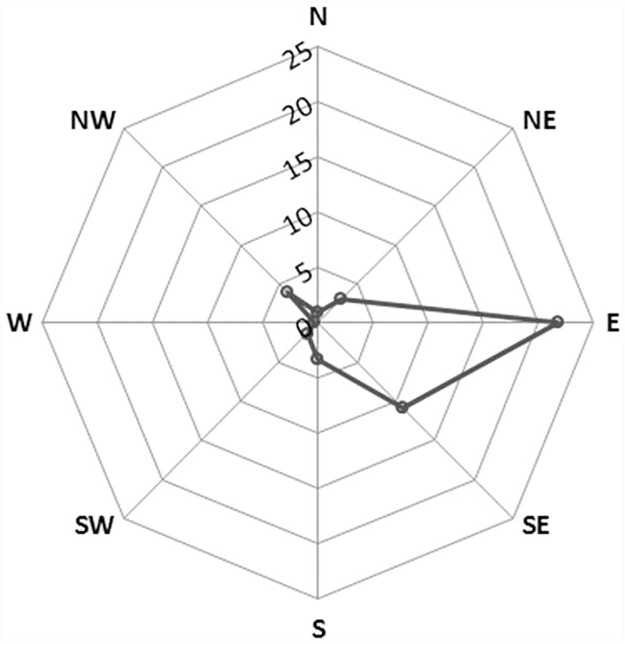
Spider plot of the anisotropic spread of the Los Angeles *D*. *citri* hotspot from 2009 to 2014. Concentric rings indicate the rate of spread in increments of 5 km per year.

Kernel density raster maps showed a clear anisotropic pattern of *D*. *citri* spread through the incremental time periods (S1 Fig). Of particular note is the observed initial hotspot of *D*. *citri* in San Diego, followed by the presence of the next hotspot for the year 2008–09 in urban Los Angeles. However, from 2008–09 onwards the spread of *D*. *citri* was continuous in both space and time as is evident from the strong eastward spread of the Los Angeles *D*. *citri* hotspot. The eastward spread of *D*. *citri* then shifts direction toward the south from 2012 onwards; thus indicating an overall east and south east spread of *D*. *citri* density from its initial strong presence in urban Los Angeles. A spider plot for the Los Angeles hotspot clarifies further that the maximal spread rate is in the eastern direction followed by the south east (Fig 9).

**Fig 10:**
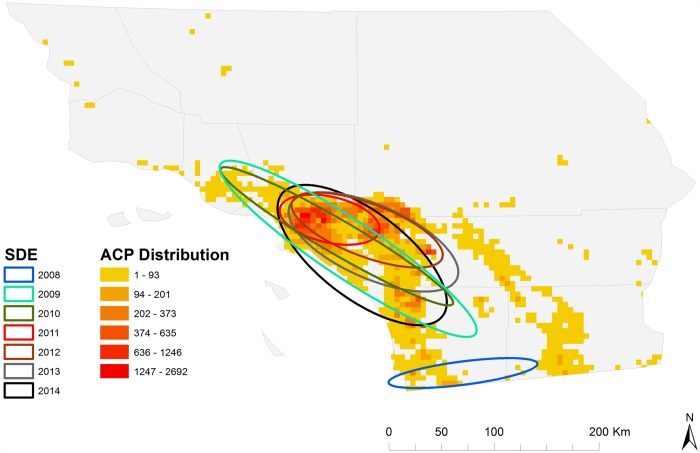
Standard deviational ellipses depicting anisotropic spread of *D*. *citri* hotspots.

The orientation of the yearly SDE further revealed the directional bias of *D*. *citri* distribution from 2008 to 2014 (Table D in S1 File). *D*. *citri* positive sites had an ellipsoid distribution during each year, with a strong east-to-west direction during 2008 in San Diego and Imperial Counties, and a stronger northwest-to-southeast directional bias during subsequent time periods in Los Angeles, Orange, Ventura, San Bernardino and Riverside Counties (Fig 10). The spatial concentration (i.e. area of each ellipse) and extent of directional dispersion (i.e. long axis of each ellipse) exhibited significant differences across years for both the distribution of *D*. *citri* positive sites and hotspots. The highest level of spatial concentration occurred in 2011 and centered on downtown Los Angeles, whereas the 2009 SDE exhibited the largest directional (northwest-to-southeast) dispersion (Fig 10). We calculated the eccentricity of each SDE (i.e. polarity of the distribution within the ellipse), which indicated significant differences in how uniformly *D*. *citri* positive sites were distributed around the mean center of each SDE. Certain years (2008, 2011) demonstrated a more polar distribution (i.e. more points distributed at each end of the ellipse with fewer in the middle). We also found significant differences between the angle of rotation (i.e. angle between north and y-axis rotated clockwise) for each year, providing further evidence of anisotropic spread of *D*. *citri* positive sites and hotspots across Southern California.

## Discussion

The impacts of invasive vector-borne diseases on ecosystem services are gaining increased attention as the far-reaching effects of global change begin to crystalize [31]. Recent increases in trade globalization have led to a rapid growth in the cross-border movement of agricultural products [32]. An unintended consequence of this trend has been the introduction of non-native insect species into a growing number of new environments [33]. An example of this emerging global threat is the recent introduction, presumptively via human transportation of infested plant material, of *D*. *citri* into Southern California in 2008 [10]. Using a spatial framework, we describe the geographic and temporal dynamics of the Southern California *D*. *citri* invasion from its presumed beginning.

We found clear evidence that the spatial distribution of *D*. *citri* in Southern California during the study period was non-random and is characterized by the presence of statistically significant hotspots of elevated entomological risk. These hotspots of *D*. *citri* abundance were strongly associated with certain urbanized regions and suggest more frequent introduction events in these areas, perhaps due to the transportation of plant material, equipment and fruit via road networks [22, 33, 34]. In addition to the transportation-mediated introduction events of invasive insects, urbanization may also be correlated with the amount of suitable habitat available for establishment and spread [35]. Subsequent spread of *D*. *citri* was likely the result of natural dispersal of the psyllid throughout areas with a high density of residential citrus trees, coupled with some continuing longer distance movement via unregulated or illegal movement of plant material [22, 33–36].

Many of the reasons behind the introduction and spread of *D*. *citri* in Southern California likely have similarities in other regions where invasion has been studied in greater detail. In Florida, where the economic burden of the *D*. *citri* invasion in the United States is greatest [14], long distance dispersal is thought to be a combination of both natural adult psyllid movement and human-mediated transportation events [16, 37]. Several studies in Florida have suggested that adult psyllids may be capable of flight distances of several kilometers and have allowed the insect to become widespread throughout the state [38–40]. Similar capabilities have also been demonstrated in Japan [41], included the purported movement of the psyllid between islands [42]. In addition to long distance migration potential, *D*. *citri* dispersal across large portions of Florida may have been further facilitated by the distribution of abandoned citrus groves [40]. Proximity to road networks and the unregulated movement of bulk citrus and infested nursery stock also contributed to the rapid distribution of *D*. *citri* in Florida [16].

Biological invasions are known to exhibit strong gradients of anisotropic spread through susceptible landscapes [43, 44]. The initial detection of *D*. *citri* in urban Los Angeles in 2009 was followed by rapid spread and infestation of urban areas in a southern and easterly direction across Los Angeles and adjoining counties of Riverside, San Bernardino and Orange, thus providing an ideal scenario to explore the observed anisotropic spread pattern. The dynamic nature of *D*. *citri* geographic distribution and evidence of anisotropic spread suggests that hotspots of entomological risk are likely a function of not only anthropogenic factors (e.g., transportation associated with trade and commerce), but also differences in environmental suitability (e.g., resource availability, temperature, wind speed, elevation) that exist across urbanization gradients [22, 33–36, 40, 41]. One explanation for the strong directional spread of the Los Angeles hot spot to the Southwest, and slower invasion to the Northwest, is that it reflects the prevailing wind direction in the area. However caution should be used in drawing this conclusion, as multiple studies from urban areas and commercial citrus in Florida have found little to no evidence that wind affects *D*. *citri* activity or dispersal ability [38, 39]. Rather, *D*. *citri*’s non-random, asymmetric spread in Southern California is likely to be primarily attributable to other aspects of habitat suitability, including warmer conditions [45–47] and more favorable landscape contexts (i.e. greater prevalence of suburban residential citrus trees) towards the interior of the region [34].

In addition to the spatial patterns observed from year-to-year, we detected modest temporal variation within years across both urban and commercial citrus sites. Seasonal variation is important to both *D*. *citri* detection and spread because the psyllid’s lifecycle and performance is tightly linked to periods of new shoot production (i.e. “flush”) that is most prominent in many California citrus groves during the spring and fall [11]. New flush production is a critical aspect of *D*. *citri* nymphal development and represents a significant component of HLB infection risk in citrus trees [48, 49]. The higher *D*. *citri* detection rate in fall months suggests that the flush of young leaves among host plants as the nights cool are key contributors to the observed seasonality pattern. This pattern was more predictable in commercial citrus sites, perhaps reflecting more substantial variability in horticultural practices among residences as compared to commercial groves. Yet, neither urban nor commercial citrus showed clear evidence of a spring peak in adult *D*. *citri* trapping frequency. This pattern is contrary to what typically occurs in Florida and Brazil [50, 51]. However, it is consistent with what has been found in other studies in California [52] and is also similar to Fall-dominance in *D*. *citri* adult trap counts that has been reported from Texas [53]. The lack of an observed spring peak is likely attributable to the impact of overwintering mortality on *D*. *citri* populations, which appears to significantly decrease *D*. *citri* population growth [54]. Colder winter temperatures may reduce *D*. *citri* numbers to below a population threshold that can adequately recover in time to fully benefit from spring flush [45–47, 55]. In addition to climatic factors, early season *D*. *citri* dynamics may be further constrained by the activity of the parasitoid *Tamarixia radiata* [17] and other resident generalist predators [54].

HLB, associated with the bacterium transmitted by *D*. *citri*, has been documented in regions throughout the world, including a recent emergence in the Western Hemisphere [10, 56, 57]. In 2012, the first HLB positive test in a hybrid-lemon citrus tree was identified in a residential neighborhood in Los Angeles County [18], confirming the relative importance of residential areas for the likelihood of HLB-related damage resulting from *D*. *citri* spread. Given the long latent period, coupled with the ability of *D*. *citri* to acquire the pathogen several months before detection in trees is possible [56, 57], it is likely that other cases exist but have yet to manifest. In regions where HLB is more widespread (i.e. China, Brazil, Florida), the spatial distribution of the disease appears clustered at the regional level and new foci of infection can be widely spaced [10]. Here we show a similar regional clustering of *D*. *citri*, suggesting that the dynamic dispersion of hotspots over time may contribute to the spread of HLB infection foci into new regions in a predictable manner.

Quantifying the spatial dynamics of entomological risk over time is a critical component of understanding invasive insect spread in human-dominated ecosystems and for prioritizing disease management objectives [58–60]. We detected hotspots of *D*. *citri* that showed clear directionally-dependent movement to the south and east after first appearing in an urbanized region of Los Angeles. This information may be useful for predicting the risk of HLB disease transmission and for further prioritizing prevention and control efforts in certain areas [60]. Specifically, disease surveillance efforts may be more effective if targeted towards the interior areas of hotspots where conditions for the establishment of *D*. *citri* are presumptively favorable and less in western areas. Further, differences in certain landscape and abiotic factors (e.g., temperature, elevation) across the region may be contributing to the observed asymmetric pattern of spread. Surveillance may also be improved by following the leading edge of hotspots along a southeastern trajectory. Given the relative stability of the Los Angeles hotspot under existing management efforts, information on the distribution of *D*. *citri* may ultimately be more useful for enhanced HLB risk prediction and less for psyllid eradication.

Currently *D*. *citri* is predominantly found in urban and suburban areas of Southern California; however, spillover into commercial citrus and areas further north has begun. The spread of *D*. *citri* and HLB in Southern California represents a significant threat to citrus in the region. To better understand this threat, we quantified the spatial distribution of entomological risk using spatial statistics to identify hot spots of *D*. *citri* abundance. Using information on the location and directional spread of hotspots as a proxy for HLB risk may be of great use towards refining disease management efforts.

## Supporting information

S1 Fig Kernel-density maps showing the anisotropic spread pattern of *D*. *citri* through incremental time periods, 2008 to 2014. (TIF)

S1 File **Table A. Yearly *Diaphorina citri* trapping statistics, by Southern California county, from two sources**. Records from the California Department of Food & Agriculture (CDFA) database most closely represents the maximum number of traps deployed at any given time over the year. Records from the United States Department of Agriculture Integrated Plant Health Information System (USDA) represent reported total number of trap deployments over the year. **Table B. Global test of spatial autocorrelation with incremental Moran’s I statistic. Table C. Six probable clusters of positive sites defined by the Kulldorff space-time permutation scan statistic, 2008–2014. Table D**. **Standard deviational ellipses of hotspot geographic center dispersion**.
(DOCX)
