# Supplementary figures and images for: Spatiotemporal dynamics of the Southern California Asian citrus psyllid (Diaphorina citri) invasion
Source: PLoS One. 2017 Mar 9;12(3):e0173226. doi: 10.1371/journal.pone.0173226 (PMC5344380; doi:10.1371/journal.pone.0173226)

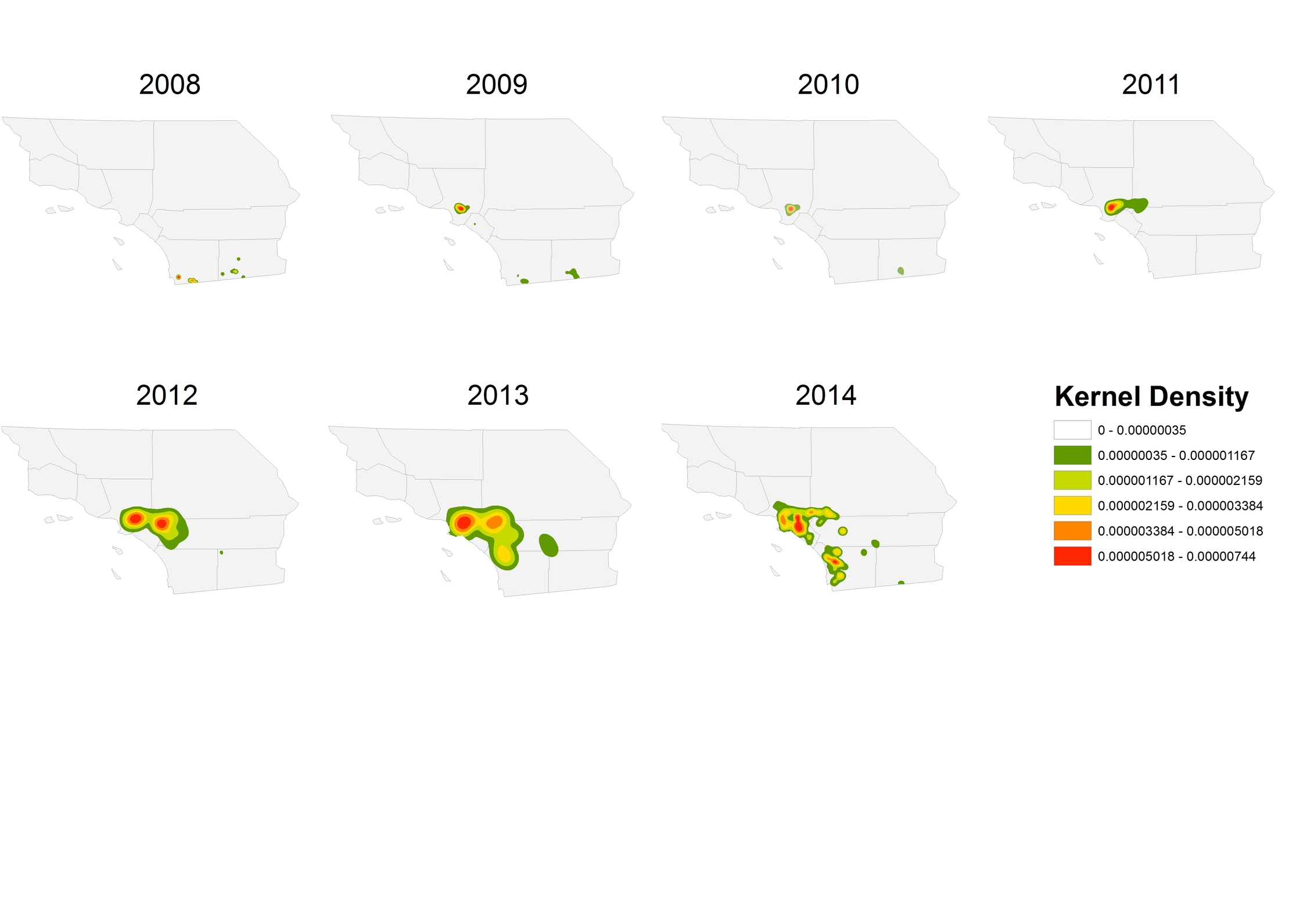

Supplement: S1 Fig — (TIF) [file pone.0173226.s001.tif]
