# Supplementary material for: Spatiotemporal dynamics of the Southern California Asian citrus psyllid (Diaphorina citri) invasion
Source: PLoS One. 2017 Mar 9;12(3):e0173226. doi: 10.1371/journal.pone.0173226 (PMC5344380; doi:10.1371/journal.pone.0173226)
Supplement: S1 File — Table A. Yearly Diaphorina citri trapping statistics, by Southern California county, from two sources. Records from the California Department of Food & Agriculture (CDFA) database most closely represents the maximum number of traps deployed at any given time over the year. Records from the United States Department of Agriculture Integrated Plant Health Information System (USDA) represent reported total number of trap deployments over the year. Table B. Global test of spatial autocorrelation with incremental Moran’s I statistic. Table C. Six probable clusters of positive sites defined by the Kulldorff space-time permutation scan statistic, 2008–2014. Table D. Standard deviational ellipses of hotspot geographic center dispersion. (DOCX) [file pone.0173226.s002.docx]

**Table A.** **Yearly *Diaphorina citri* trapping statistics, by Southern California county, from two sources.** Records from the California Department of Food & Agriculture (CDFA) database most closely represents the maximum number of traps deployed at any given time over the year. Records from the United States Department of Agriculture Integrated Plant Health Information System (USDA) represent reported total number of trap deployments over the year.

|  | **2008** | | **2009** | | **2010** | | **2011** | | **2012** | | **2013** | | **2014** | |
| --- | --- | --- | --- | --- | --- | --- | --- | --- | --- | --- | --- | --- | --- | --- |
| **County** | **CDFA** | **USDA** | **CDFA** | **USDA** | **CDFA** | **USDA** | **CDFA** | **USDA** | **CDFA** | **USDA** | **CDFA** | **USDA** | **CDFA** | **USDA** |
| Imperial | 1452 | 1 | 3794 | 854 | 6535 | 895 | 4423 | 78980 | 4358 | 83999 | 5683 | 23597 | 487 | 39856 |
| Kern | 0 | -- | 0 | -- | 1205 | -- | 1400 | -- | 1851 | 81823 | 1865 | 97381 | 2907 | 25546 |
| Los Angeles^1^ | -- | -- | 9032 | -- | 16623 | -- | 13953 | -- | 2673 | -- | 100 | -- | 100 | -- |
| Orange | 1008 | -- | 2119 | -- | 2431 | 238 | 1754 | 6656 | 824 | 20809 | -- | 8 | 0 | -- |
| Riverside | --^2^ | -- | 3480 | -- | 4686 | -- | 5041 | -- | 1411 | 44383 | -- | 54403 | 12 | -- |
| San Bernardino | -- | -- | 998 | -- | 2838 | 409 | 2075 | 7019 | 1060 | 19297 | -- | -- | 15 | -- |
| San Diego | 2310 | -- | 9581 | -- | 5477 | 34 | 3428 | 3887 | 2168 | 42087 | 2488 | 41795 | 133 | -- |
| San Luis Obispo | 0 | -- | 0 | -- | 1263 | -- | 1250 | -- | 400 | 12851 | 433 | 43709 | 1388 | 88091 |
| Santa Barbara | 0 | -- | 0 | -- | 26 | -- | 410 | 2110 | 824 | 3964 | 815 | 3529 | 342 | 6971 |
| Ventura | -- | -- | 525 | -- | 1420 | -- | 4255 | -- | 2914 | -- | 3679 | 807 | 2110 | 74 |
| *Yearly total* | 4770 | 1 | 29529 | 854 | 42504 | 1576 | 37989 | 98652 | 18483 | 309213 | 15063 | 265229 | 7494 | 160538 |
| ^1^No trapping statistics are available in the USDA-IPHIS database for Los Angeles County  ^2^No trapping statistics are reported in the database although some trapping may have been occurring | | | | | | | | | | | | | | |

**Table B.** **Global test of spatial autocorrelation with incremental Moran’s I statistic.**

| **Year** | **Distance (m)** |
| --- | --- |
| 2008 | 6,000 |
| 2009 | 9,000 |
| 2010 | 10,000 |
| 2011 | 14,000 |
| 2012 | 24,000 |
| 2013 | 30,000 |
| 2014 | 12,000 |

**Table C. Six probable clusters of positive sites defined by the Kulldorff space-time permutation scan statistic, 2008-2014.**

| **Cluster** | **Period** | **RR** | **Test statistic** | **P-value** |
| --- | --- | --- | --- | --- |
| 1 | 2009-2011 | 4.08 | 4109.37 | <.001 |
| 2 | 2014 | 3.67 | 2115.73 | <.001 |
| 3 | 2014 | 3.02 | 2000.88 | <.001 |
| 4 | 2012 | 2.06 | 1683.60 | <.001 |
| 5 | 2008-2010 | 7.16 | 1292.99 | <.001 |
| 6 | 2011 | 2.06 | 726.53 | <.001 |

**Table D**. **Standard deviational ellipses of hotspot geographic center dispersion.**

| **Year** | **X Std. Distance** | **Y Std. Distance** | **Eccentricity** | **Rotation** |
| --- | --- | --- | --- | --- |
| 2008 | 12288.84 | 70491.36 | 0.17 | 83.09 |
| 2009 | 140550.68 | 25982.82 | 5.41 | 124.28 |
| 2010 | 119726.97 | 13871.35 | 8.63 | 120.28 |
| 2011 | 13880.91 | 39334.86 | 0.35 | 85.75 |
| 2012 | 56241.85 | 23029.28 | 2.44 | 102.54 |
| 2013 | 67696.96 | 36179.80 | 1.87 | 114.95 |
| 2014 | 85228.86 | 37761.41 | 2.26 | 132.54 |
